# ^18^F-choline PET/CT and PET/MRI in primary and recurrent hyperparathyroidism: a systematic review of the literature

**DOI:** 10.1007/s12149-020-01507-1

**Published:** 2020-08-07

**Authors:** Laura Evangelista, Ilaria Ravelli, Fabio Magnani, Maurizio Iacobone, Chiara Giraudo, Valentina Camozzi, Alessandro Spimpolo, Diego Cecchin

**Affiliations:** 1 Nuclear Medicine Unit, Department of Medicine (DIMED), University of Padova, Via Giustiniani 2, 35128 Padua, Italy; 2 Surgery Unit, Department of Surgery, University of Padova, Padua, Italy; 3 Radiology Unit, Department of Medicine (DIMED), University of Padova, Padua, Italy; 4 Endocrine Unit, Department of Medicine (DIMED), University of Padova, Padua, Italy; 5 International PhD Program in Arterial Hypertension and Vascular Biology (ARHYVAB), Department of Medicine (DIMED), University of Padova, Padua, Italy

**Keywords:** 18F-choline, Hyperparathyroidism, PET/CT, PET/MRI, Systematic review

## Abstract

The aims of the present systematic review were to: (1) assess the role of ^18^F-fluorocholine (FCH) positron emission tomography (PET) with computed tomography (CT) and PET with magnetic resonance imaging (MRI) in patients with biochemically known hyperparathyroidism; (2) compare the diagnostic performance of FCH PET/CT or PET/MRI with conventional morphological and functional imaging. A literature search until December 2019 was performed in the PubMed, Scopus and Web of Science databases, using the terms “choline” AND “PET” AND “hyperparathyroidism”. The search was conducted with and without the addition of filters (e.g., language: English only; type of article: original article; subjects: humans only) and selecting only articles published in the last 5 years. Twenty-three articles and 1112 patients were considered. Different FCH PET/CT acquisition protocols were adopted across the studies, using dynamic, early or delayed scans. FCH PET/CT proved more accurate than ultrasonography (US) or 99mTc-sestamibi single-photon emission tomography (SPET). PET/MRI also seemed to be more accurate than MRI alone in detecting benign parathyroid lesions. FCH PET/CT is more accurate than conventional morphological and functional imaging modalities (US or SPET) for the detection of benign parathyroid lesions. It could, therefore, be a reliable tool in both primary and recurrent hyperparathyroidism.

## Introduction

Primary hyperparathyroidism (PHPT) is a rather common endocrinological disorder, the third most common endocrine disease after diabetes mellitus and thyroid disorders. It is characterized by one or more hyperfunctioning parathyroid glands [1], due to parathyroid adenoma (in > 80% of cases), multiple adenomas, parathyroid hyperplasia (about 15%) or parathyroid carcinoma (less than 1%) [2], [3]. People with mild PHPT are at increased risk of various comorbidities, such as nephrolithiasis, osteoporosis and fragility fractures. Surgical excision is usually a definitive treatment, though repeat surgery may be required in cases of recurrent or persistent hyperparathyroidism (fewer than 5% of patients) [4].

PHPT may also be associated with a higher risk of cardiovascular disease and mortality [5, 6]. The related mortality risk seems to be lower in patients who undergo surgery than in those treated conservatively [6]. A recent cost-effectiveness analysis on patients with non-localized PHPT showed that use of advanced imaging methods is more cost-effective than routine bilateral neck exploration [6]. Based on this evidence, it becomes essential to employ appropriate methods capable of revealing which parathyroid glands are causing PHPT. This is also important to avoid unnecessary surgery and to identify ectopic parathyroid glands.

Benign parathyroid lesions can be identified and located using morphological or functional imaging. Neck ultrasonography (US), computed tomography (CT) and magnetic resonance imaging (MRI) have been extensively used in this setting. Dual-tracer subtraction, mainly using 99mTc-sestamibi (MIBI)/99mTc-pertechnetate scans, and/or dual-phase scintigraphy with planar acquisitions, have been widely employed in cases of hyperparathyroidism, frequently combined with US. For diagnostic purposes, single-photon emission tomography (SPET)/CT has proved the best imaging modality for identifying the site of hyperfunctioning parathyroid glands, better than either SPET alone or planar scintigraphy. That said, even SPET/CT fails to identify the gland in up 30% of cases [7].

When a combination of US and SPET/CT fails, a more effective imaging technique is needed. Radiolabeled choline PET/CT has been widely used in patients with recurrent prostate cancer [8], and it was in this context that a study published in 2013 reported incidentally detecting an 18F-fluorocholine (FCH) hotspot in a patient’s neck, which turned out to be a parathyroid adenoma [9]. In the ensuing 6 years, a number of published studies reported on the role of FCH PET/CT in identifying benign parathyroid lesions.

The aims of the present systematic review are to: (1) assess the role of FCH PET/CT and PET/MRI in patients with known PHPT; and (2) compare the diagnostic performance of FCH PET/CT or PET/MRI with other morphological and/or functional imaging modalities.

## Materials and methods

### Search strategy and study selection

A literature search until December 2019 was performed in the PubMed, Scopus and Web of Science databases. The terms used were: “choline” AND “PET” AND “hyperparathyroidism”. The search was run with and without the addition of filters, such as language (English only), type of article (original article), subjects (humans only), and only considering articles published in the last 5 years. Two reviewers (I.R., F.M.) conducted the literature search, and two independent physicians (L.E., D.C.) selected studies for inclusion, and data extraction. Any discrepancy was resolved by consensus. Independently identified records were combined, then the full texts were retrieved and examined by three reviewers (I.R., F.M., L.E.). The reference lists of the selected studies were carefully checked to identify any additional relevant literature.

A systematic review was conducted using established methods [10], and the results are presented according to the PRISMA guidelines [11].

Only studies that met the following inclusion criteria were considered eligible for the systematic review: (a) a sample size of more than 10 patients; and (b) FCH PET/CT as the index test. Clinical reports, conference abstracts, and editors’ comments were excluded. Systematic reviews, with or without meta-analyses, were considered for the purpose of enriching the bibliographic references.

### Data extraction

For each study considered, the general information retrieved included: basic data (authors, year of publication, country, and study design); population characteristics (number of patients, type of hyperparathyroidism, PTH levels); the diagnostic reference standard; and the comparison of FCH PET/CT with other imaging modalities (i.e., US, 99mTc-MIBI SPET, or SPET/CT).

### Statistical analysis

Continuous variables were expressed as median (range) and categorical as number (percentage). Data about diagnostic accuracies were obtained from each study to prepare a 2 × 2 contingency table and thus calculating the pooled sensitivity, by a patient-based and lesion-based analysis. Comprehensive meta-analysis (CMA) software version 3.3.070 (Biostat, Englewood, NJ, USA) was used for the assessment of pooled diagnostic performances.

## Results

**Fig. 1:**
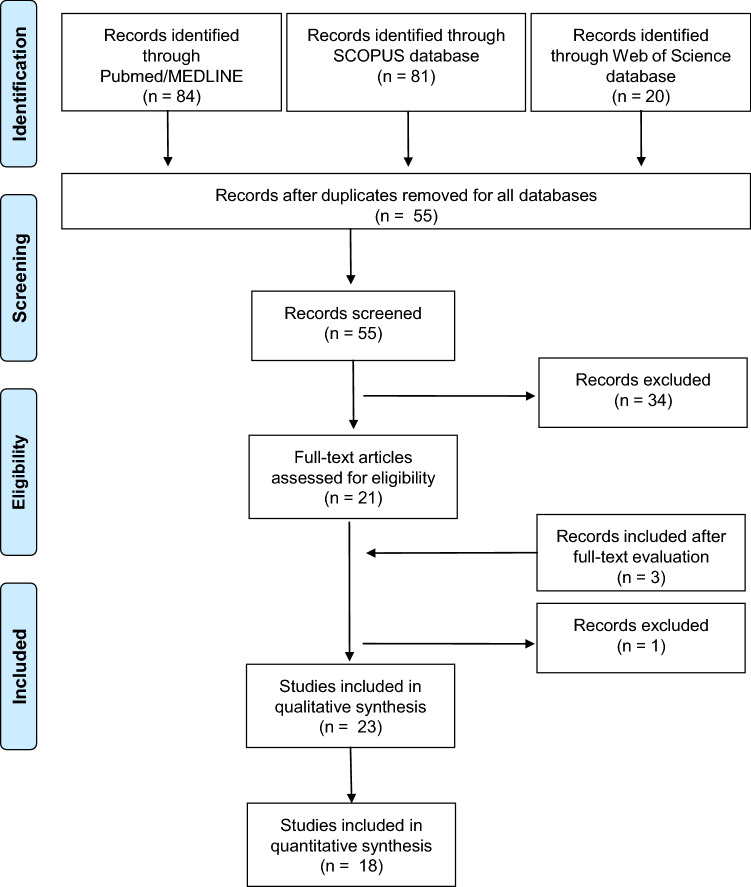
PRISMA flow for the selected studies

The systematic literature search generated 23 articles (see Fig. 1) concerning a total of 1112 patients investigated with FCH PET/CT for the detection of benign parathyroid lesions. Most of the studies were prospective (*n* = 15, 65%; 488 patients). Interestingly, 67 patients underwent FCH PET/MRI. As shown in Table 1 [12–33], FCH PET/CT was compared in many cases with US or 99mTc-MIBI scanning, the main endpoint being to assess the accuracy of FCH PET/CT in detecting benign parathyroid lesions in cases of doubtful or negative conventional imaging. In all studies, histopathological examination was the diagnostic reference standard.

**Table 1:** Summary of selected studies ordered by acquisition type (Dynamic or static with single or double time points) and year of publication

Authors, ref	Year pub	Country	N of pts	Study design	PTH level Median (range) Mean ± SD	Type of parathyroid disease	Protocol FCH PET (dosage)	End-point	Comparison with other modalities	Outcome
*Dynamic studies*										
Michaud et al. [12]	2014	France	12	P	39.4 (4–176) ng/mL	PHPT and SHPT	Dyn.10 min + Stat. (3 MBq/kg)	To check if FCH uptake was a general feature of adenomatous or hyperplastic parathyroid glands	US and/or 123I/MIBI dual-phase scintigraphy	PTH decrease from 60 to 95%.
Michaud et al. [13]	2015	France	17	P	280 (61–1946) pg/mL	PHPT and SHPT	Dyn. 10 min (no activity data)	To compare FCH-PET/CT findings in pts with discordant or equivocal results at US and scintigraphy	US and/or I123 + MIBI scintigraphy	FCH-PET/CT sensitivity is better than that of US and not inferior to that of dual-phase I123 + sestaMIBI scintigraphy
Kluijhout et al. [14]	2017	US	10	P	86 ± 43 ng/mL	PHPT	Dyn. 40 min (no activity data)	To investigate the performance of FCH PET/MR imaging in patients with HP and non-localized disease who have negative or inconclusive results at US and MIBI scintigraphy	US, MIBI SPET/CT	FCH PET/MR imaging allowed localization of adenomas with high accuracy when conventional imaging results were inconclusive and provided detailed anatomic information.
Prabhu et al. [15]	2018	India	14	P	NA	PHPT and PTA	Dyn. 15 min + Stat. 45-60 min (185-296 MBq)	To assess the utility of early dynamic FCH PET/CT in detecting parathyroid lesions and differentiating parathyroid lesions from cervical lymph nodes	None	Early dynamic FCH PET/CT can detect parathyroid adenomas in PHPT
*Static studies (single time point)*										
Kluifhout et al. [16]	2016	The Netherlands	44	R	NA	HPT (MEN 1 and hyperpl.)	Stat. 30 min (2 MBq/Kg)	FCH PET/CT performance as second line imaging scan	None	FCH PET/CT is able to identify a HPT in case of inconclusive US and sestamibi
Quak et al. [17]	2017	France	25	P	94.8 ± 37.4 ng/mL	PHPT and PTA	Stat. 60 min (1.5 MBq/kg)	Evaluate the sensitivity of FCH PET/CT for PTA detection prior to surgery in patients with PHPT and negative or inconclusive cervical ultrasound and MIBI SPET/CT	US and MIBI SPET/CT	88% patients were considered cured after surgery
Grimaldi et al. [18]	2018	France	27	P	102.5 (59.0-514.0) ng/mL	PHPT	Stat. 30 min (100 MBq)	To evaluate the added value of pre-surgical FCH-PET/CT in localizing hyperfunctioning parathyroid glands	US, MIBI + Tc SPET/CT	FCH-PET/CT is a promising modality in challenging pre-surgical localization
Huber et al. [19]	2018	Switzerland	26	R	110.8 (54.9-257.6) ng/mL	PHPT	Stat. 10 min (150 MBq)	FCH-PET/CT or MRI ability to pre-surgical localization of PT in case of negative or conflicting US and scintigraphy	US, I123 + Tetrofosmin SPET/CT	FCH-PET is a highly accurate method to detect PT adenomas even in case of failure of other imaging examinations
Araz et al. [20]	2018	Turkey	35	P	123.06 ± 34.82 ng/mL	PHPT	Stat. 45-60 min (100 MBq)	Comparison between FCH PET/CT and MIBI SPET/CT in hyperparathyroidism and the utility of SUVmax for the evaluation of disease severity	MIBI SPET/CT	FCH has a higher performance than MIBI SPECT/CT. SUV is correlated with PTH and bone mineral densitometry (BMD) scores
Piccardo et al. [21]	2018	Italy	44	P	120.7 (71.8–545) ng/mL	PHPT	Stat. 10 min (100 MBq)	Comparison among integrated FCH-PET/4DCeCT and FCH-PET/CT and 4DCeCT detection rate and sensitivity	4DCeCT	Integrated FCH-PET/4DCeCT has a performance superior to that of FCH-PET/CT and 4DCeCT, separately
Zajickova et al. [22]	2018	Czech Republic	13	P	114.6 (78.9–145) ng/mL	PHPT	Stat. 30 ± 20 min (180 MBq)	FCH PET/CT was performed after inconclusive neck US and MIBI SPET scintigraphy in patients with PHPT to localize abnormal parathyroid glands before surgery	US and MIBI scintigraphy	FCH correctly identified PTA and hyperplastic glands in 92% patients with previously inconclusive conventional imaging
Fischli et al. [23]	2018	Switzerland	39	R	168.39 ± 110.69 ng/mL	PHPT	Stat. 45 min (160 MBq)	To evaluate the sensitivity and specificity of FCH-PET/CT for preoperative localization in patients with pHPT and negative or equivocal 99mTc-sestamibi scintigraphy and/or ultrasound	None	FCH-PET/CT provides an excellent sensitivity of > 90% per-patient and of > 87% per lesion-based level
Amadou et al. [4]	2019	France	29	R	122.81 ± 50.78 ng/mL	PHPT	Stat. 60 min (230 MBq)	To evaluate FCH-PET/CT and parathyroid 4D-CT as to guide surgery in patients with PHPT and prior neck surgery	US, MIBI scintigraphy and/or MIBI SPET/CT, 4D-CT	Superiority of FCH-PET/CT and 4D-CT compared to first-line imaging in re-operative patients
***Static studies (Dual Time Point):***										
Lezaic et al. [24]	2014	Slovenia	24	P	NA	PHPT	Stat. 5 and 60 min (100 MBq)	Evaluate the usefulness of FCH PET/CT for preoperative localization of hyperfunctioning parathyroid tissue	MIBI SPET/CT, MIBI dual-phase and MIBI/Tc subtraction imaging	FCH PET/CT appears to be a promising, effective imaging method for localization of hyperfunctioning parathyroid tissue
Rep et al. [25]	2015	Slovenia	43	P	311.5 (70.6–2022) ng/mL	PHPT	Stat 5, 60 and 120 min (100 MBq)	To determine the optimal scan time, i.e., time between radiopharmaceutical administration and FCH PET/CT imaging in patients with a PHPT	Conventional MIBI scan	Optimal imaging time is one hour after the administration of FCH
Hocevar et al. [26]	2016	Slovenia	151	R	NA	PTA	Stat. 5 and 60 min (100 MBq)	To analyze the results of FCH-PET/CT pre-surgical localization and the possibility to skip ioPTH testing in pts with single adenoma	US, MIBI SPECT/CT	FCH-PET/CT is a reliable test in pre-surgical localization and pts with single PTA on PET can safely undergo a focused parathyroidectomy
Rep et al. [27]	2018	Slovenia	36	P	NA	PHPT	Stat. 5 and 60 min (100 MBq)	To measure the organ doses and the ED for conventional subtraction parathyroid imaging protocols, using dual-phase MIBI SPET/CT as a potential conventional imaging method of choice and FCH dual-phase PET/CT as a potential future imaging method of choice for localisation of HPGs	Parathyroid subtraction scintigraphy and dual-phase SPET/CT	In HPGs, SPET/CT and PET/CT have a superior diagnostic performance than conventional scintigraphy
Alharbi et al. [28]	2018	Switzerland	52	R	122.4 ± 49.9 ng/mL	PTA (only single adenomas)	Stat. 2 and 50 min (150 MBq)	To investigate the relationship between FCH-PET (MR and CT) results and PTH levels	None	FCH uptake in PTA is strongly correlated with preoperative PTH serum levels
Beheshti et al. [29]	2018	Austria	100	P	196.5 ± 236.4 pg/mL	PTA	Stat. 60 and 120 min (3.2 MBq/Kg)	To compare assessment of PHPT from FCH-PET/CT and MIBI or Tetrofosmin SPECT/CT	MIBI or Tetrofosmin SPECT/CT	FCH-PET/CT is clearly superior to MIBI/Tetrofosmin SPET/CT in detecting PTA, especially small ones
Bossert et al. [30]	2018	Italy	34	P	179.9 ± 123.1 ng/mL (Hypercalcemic) 158.4 ± 55.4 ng/mL (Normocalcemic)	PTA	Stat. 9 and 60 min (3.5 MBq/Kg MBq)	To compare diagnostic performance of FCH-PET/CT with MIBI + TC SPET/CT	US, MIBI + TC SPET/CT	FCH-PET/CT can be considered a first line imaging technique in pts with normo- or hypercalcemic PHTP
Christakis et al. [31]	2019	UK	12	R	19.1 ± 5.11 ng/mL	PHPT or recurrent PTA	Stat. 60 and 90 min (300 MBq)	To assess if FCH PET/CT is able to identify parathyroid adenomas, with a negative scan	None	FCH PET/CT is able to identify the presence of adenoma parathyroid also in case of negative conventional imaging
Thanseer et al. [32]	2019	India	54	P	165.5 (117–362.5) ng/mL in eutopic 302 (236–1264) ng/mL in ectopic	PHPT	Stat. 10-15 min + 60 min (150-185 MBq)	to compare pre-surgery localization in US, MIBI SPECT/CT and FCH PET	US, MIBI SPECT/CT	FCH PET/CT has higher sensitivity and specificity especially in patients with small and ectopic PHPT and low, slight PTH values
Broos et al. [33]	2019	The Netherlands	271	R	16.1 ± 11.3 ng/mL	PHPT	Stat. 5 and 60 min (150 MBq)	To evaluate FCH PET/CT as a first-line modality	None	High detection rates of FCH PET/CT in PHPT. FCH PET/CT can be used as a first-line imaging modality in preoperative planning of parathyroid surgery

*SD* standard deviation, *PHPT* primary hyperparathyroidism, *SHPT* secondary hyperparathyroidism, *PTA* parathyroid adenoma, *Stat*. Static acquisition, *Dyn*. Dynamic acquisition, *P* Prospective study, *R* Retrospective study, *NA* not available

A careful analysis of the selected literature showed that a dynamic FCH PET/CT protocol was used in 4/23 studies. In particular, Michaud et al. [13] and Kluijhout et al. [14] ran a single dynamic acquisition lasting 10 min and 40 min, respectively; and Michaud et al. and Prabhu et al. [12, 15] performed a dynamic acquisition for 10-15 min followed by a static image 10 or 45 min after the injection. Static acquisitions were obtained in the other studies at a single point ranging from 10 to 60 min after the intravenous administration of FCH in 9/23 studies [4, 16, 17, 19–23, 34]; and twice, first after 2–60 min and then after 60–120 min, in 10/23 studies. Only Rep et al. [25] reported scanning patients 3 times, at 5, 60 and 120 min after FCH injection. The FCH dosage was fixed in 16 studies, in the range of 100-230 MBq [15, 19–28, 31–34]; it was adjusted to the patient’s body weight in 5 (1.5–3.2 MBq/kg) and not declared in two studies [12, 16, 17, 29, 30].

FCH PET/CT was compared with US by Amadou et al. [4], Bossert et al. [30], Hocevar et al. [26], Michaud et al. [13], and Thanseer et al. [32]. FCH PET/CT proved to be superior to US in detecting benign parathyroid lesions, with a sensitivity in the range of 85.2–100% and 50–82%, respectively, on patient-based and lesion-based analysis.

Comparisons between FCH PET/CT and 99mTc-MIBI SPET/CT with a dual-phase and/or subtraction protocol were reported in numerous studies (Table 2; [4, 12–17, 19–34]).

**Table 2:** Comparative performance of FCH PET/CT or PET/MRI Vs conventional imaging modalities ordered by acquisition type (Dynamic or static with single or double time points) and year of publication

Authors, ref	Stand. of ref.	FCH PET/CT					Comparative imaging				
		SENS.	SPEC.	PPV	NPV	ACC.	SENS.	S SPEC.	PPV	NPV	ACC.
*Dynamic studies*											
Michaud et al. [12]	Histology	89% (PL)		94% (PL)							
Michaud et al. [13]	Histology	94% (PP) 96% (PL) both in masked and open reading	PL: 88% (open) 56% (masked)	–	–	PL: 94% (open) 85% (masked)	US: 50% (PP) 50% (PL) I123 + SestaMIBI: 94% (PP) 83% (PL) both in masked and open reading	PL: 33% (US) 56% (I123 + SestaMIBI) both in masked and open reading	–	–	PL: 46% (US) 76%(I123 + SestaMIBI) both in masked and open reading
Kluijhout et al. [14]	Histology	90%		100%							
Prabhu et al. [15]	Histology	–	–	–	–	–	–	–	–	–	–
*Static studies (single time point)*											
Klujfhout et al. [16]	Histology	97.1%	–	97.1%	–	97.1%	–	–	–	–	–
Quak et al. [17]	Histology	91.3% (PL) 90.5% (PP)	–	87.5% (PL) 86.4% (PP)	–	80.7% (PL)	–	–	–	–	–
Grimaldi et al. [18]	Histology and follow up	81% (PP) 76% (PL)	– 91% (PL)	94% (PP) 85% (PL)	– 86% (PL)	– –	–	–	–	–	–
Huber et al. [19]	Histology and biochemistry	96.2% (PP)	–	100% (PL)	–	–	–	–	–	–	–
Araz et al. [20]	Follow-up and histology	96%	100%	100%	93%	97%	78%	100%	100%	70%	86%
Piccardo et al. [21]	Histology, biochemistry and follow up	81% (PL)	–	–	–	–	PL: 54.5% (4DCeCT) 100% (PET + 4DCeCT)	–	–	–	–
Zajickova et al. [22]	Histology	92%		100%		92%					
Fischli et al. [23]	Histology	95.5% (PP) 87.5% (PL)	–	–	–	–	–	–	–	–	–
Amadou et al. [4]	Histology and follow up	85.2% (PP) 95.8% (PL)	12.5% (PL)	76.7% (PL)	50% (PL)	–	PL: 54.2% (US) 50% (MIBI) 75% (4D-CT)	PL: 75% (US) 75% (MIBI) 40 (4D-CT)	PL: 86.7% (US) 85.7% (MIBI) 80% (4D-CT)	PL: 35.3% (US) 33.3% (MIBI) 33.3% (4D-CT)	–
*Static studies (dual time point)*											
Lezaic et al. [24]	Histology	92%	100%	100%	96%	98%	49% (MIBI SPET/CT) 46% (MIBI-Tc) 44% (MIBI dual-phase)	100% (MIBI SPET/CT, MIBI-Tc and MIBI dual-phase)	100%	80%	83%
Rep et al. [25]	Histology	90.5% (5 min) 93.6% (1 h) 93.6% (2 h) 95.3% (all)	98.2% (5 min) 98.2% (1 h) 98.2% (2 h) 98.2% (all)	96.6% (5 min) 96.7% (1 h) 96.7% (2 h) 96.8% (all)	94.7% (5 min) 96.4% (1 h) 96.4% (2 h) 97.3% (all)	94.1% (5 min) 96.5% (1 h) 96.5% (2 h) 97% (all)	–	–	–	–	–
Hocevar et al. [26]	Histology and biochemistry	–	–	95.2% (PL) 96.8% (per single PTA)	–	–	61% (US per single PTA) 62% (MIBI-SPET/CT per single PTA)	–	–	–	–
Rep et al. [27]	Histology	97%	99%				46% (PSS), 64% (SPET/CT)	98% (PSS), 96% (SPET/CT)			
Alharbi et al. [28]	PET positivity and histology	–	–	–	–	–	–	–	–	–	–
Beheshti et al. [29]	Histology and follow up	93.7% (PL)	96% (PL)	90.2% (PL)	97.4% (PL)	95.3% (PL)	60.8% (PL)	98.5% (PL)	94.1% (PL)	86.3% (PL)	87.7% (PL)
Bossert et al. [30]	Histology or citology/biochemistry	88%	–	–	–	–	82% (US) 17% (Tc + MIBI SPET/CT)	–	–	–	–
Christakis et al. [31]	histology	58.3%	–	–	100%	58.3%	–	–	–	–	–
Thanseer et al. [32]	Histology	100% (PP) 100% (PL)	– –	96.3% (PP) 92.8% (PL)	– –	96.3% (per pts) 92.8% (PL)	MIBI SPECT/CT 80.7% (PP) 76.4% (PL) US 69.3% (PP) 69.3% (PL)	MIBI SPECT/CT 100% (PP) 75% (PL) US 29% (PL)	MIBI SPECT/CT 97.7% (PP) 97.7% (PL) US 87.1% (PP) 87.1% (PL)	MIBI SPECT/CT 9% (PP) 23% (PL) US 12% (PL)	MIBI SPECT/CT 79.6% (PP) 80.4% (PL) US 62.9% (PP) 64.3% (PL)
Broos et al. [33]	Histology	96% (PP) 90% (PL)		100%		96% (PP) 90% (PL)					

*SENS*. sensitivity, *SPEC*. specificity, *ACC*. accuracy, *PP* per patient, *PL* per lesion

Quak et al. [17], Araz et al. [20], Kluijhout et al. [16], Hocevar et al. [26], and Thanseer et al. [32] found FCH PET/CT more sensitive than dual-phase SPET/CT (100% vs. 80.7%, and 100% vs. 76.4%, respectively, for patient-based and lesion-based analyses).

Michaud et al. [12, 13], Lezaic et al. [24], Zajickova et al. [22], Rep et al. [27], Amadou et al. [4], Beheshti et al. [29], Bossert et al. [30], Grimaldi et al. [34], and Huber et al. [19] compared FCH PET/CT with 99mTc-MIBI/99mTc-tetrofosmin SPET/CT performed with both subtraction and dual-phase protocols. They found FCH PET/CT superior to SPET/CT with the subtraction and dual-phase protocols for the detection of adenoma and/or hyperplastic parathyroid, with a diagnostic accuracy of 97.4% and 87.7% for PET/CT and SPET/CT, respectively, on lesion-based analyses.

**Fig. 2:**
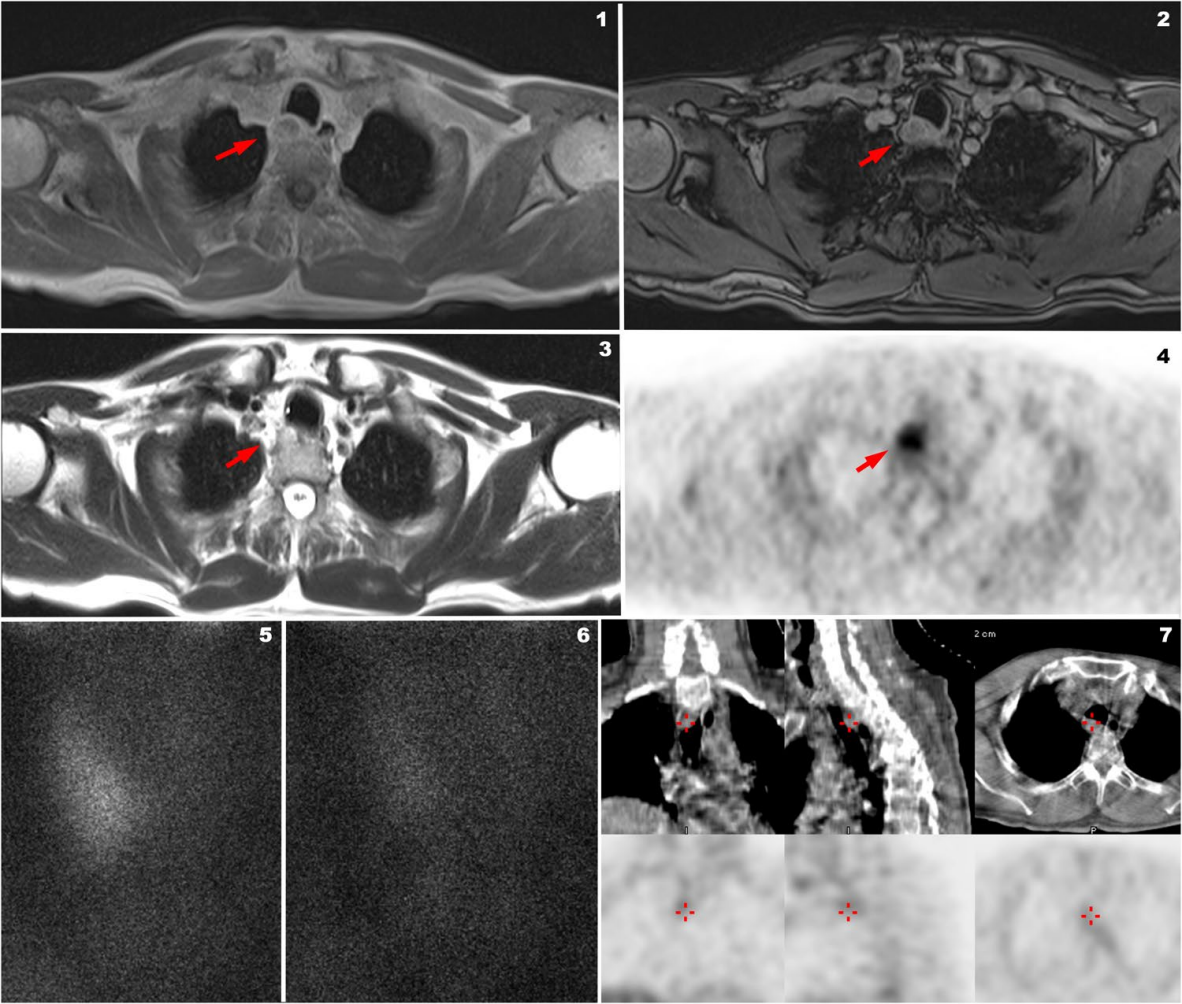
Example of positive 18F-FCH PET/MRI (Biograph mMR, Siemens Germany) and negative 99MTc-MIBI SPECT/CT (Infinia Hawkeye, GE Healthcare) performed at the University Hospital of Padova, Department of Medicine, Unit of Nuclear Medicine in a patient with hyperparathyroidism after left thyroidectomy (during a surgical procedure of left parathyroidectomy). MR axial Caipirinha in-phase (1) and MR axial Caipirinha out of-phase (2) demonstrating an ovoidal mass (red arrows) partially liquid at axial T2-Haste (3) with a very high posterior, paratracheal uptake of 18F-FCH PET/MR (4, red arrow). 99MTc-MIBI early after injection (5) and late after injection (6) phases of the same patient demonstrating no significant late retention of MIBI in the area revealed by 18F-FCH PET/MRI. SPET/CT of the same patient revealed only faint uptake (7: red cross) of MIBI in the paratracheal area revealed by 18F-FCH PET comparable to the background

In the study by Kluijhout et al. [14], 10 patients were studied with FCH PET and MRI, and compared with the same patients examined using US and a dual-phase 99mTc-MIBI SPET/CT. While for PET/MRI the sensitivity was 90% and the positive predictive value (PPV) was 100%, MRI alone showed a sensitivity of 55.6% and a PPV of 83.3%. In Fig. 2 is reported a 65-year-old patient with persistent hyperparathyroidism after surgery who underwent FCH PET/MRI in our Department.

**Fig. 3:**
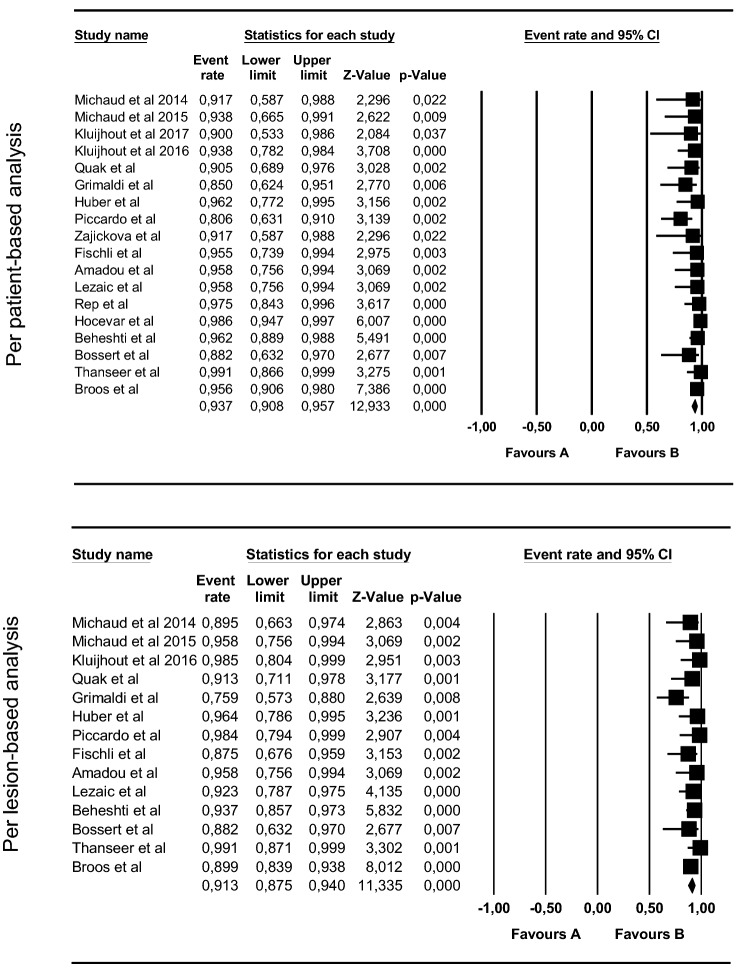
Forest plots for pooled sensitivities of FCH PET/CT or PET/MRI per patient-based and lesion-based analysis

True positive, false positive, true negative and false positive were available in 18 studies, at patient-based analysis and in 14 studies at lesion-based analysis (Table 3). Totally, true positive findings were reported in 686 patients and 530 lesions, respectively. Conversely, the number of false negative results were 35 and 23 on patient-based and lesion-based analysis. Pooled sensitivities were 93.7% and 91.3%, on patient-based and lesion-based analysis, respectively, as illustrated in Fig. 3.

**Table 3:** Diagnostic data of FCH PET/CT or PET/MRI, based on patient-based and lesion-based analysis

Authors, ref	Year publ.	Patient-based analysis					Lesion-based analysis				
		*N*	TP	TN	FP	FN	*N*	TP	TN	FP	FN
Michaud et al. [12]	2014	12	11	0	0	1	20	17	0	1	2
Lezaic et al. [24]	2014	24	23	0	0	1	39	36	0	0	3
Michaud et al. [13]	2015	16	15	0	0	1	25	23	0	1	1
Klujfhout et al. [16]	2016	33	30	0	1	2	35	33	1	1	0
Kluijhout et al. [[14]	2017	10	9	0	0	1	–	–	–	–	–
Fischli et al. [23]	2017	23	21	0	1	1	29	21	4	1	3
Hocevar et al. [26]	2017	151	144	1	4	2	–	–	–	–	–
Thanseer et al. [32]	2017	54	52	0	2	0	58	54	0	4	0
Quak et al. [17]	2018	24	19	0	3	2	26	21	0	3	2
Grimaldi et al. [18]	2018	21	17	0	1	3	76	22	43	4	7
Huber et al. [19]	2018	26	25	0	0	1	28	27	0	0	1
Zajickova et al. [22]	2018	13	11	0	1	1	–	–	–	–	–
Rep et al. [25]	2018	144	39	103	1	1	–	–	–	–	–
Beheshti et al. [29]	2018	82	76	3	0	3	277	74	190	8	5
Piccardo et al. [21]	2019	31	25	0	0	6	31	31	0	0	0
Amadou et al. [4]	2019	25	23	1	0	1	32	23	1	7	1
Bossert et al. [30]	2019	17	15	0	0	2	17	15	0	0	2
Broos et al. [33]	2019	137	131	0	0	6	148	133	0	0	15

*TP* true positive, *TN* true negative, *FP* false positive, *FN* false negative

## Discussion

The present systematic review showed that, in a population with negative/doubtful imaging findings, FCH PET/CT is more accurate than 99mTc-MIBI scintigraphy (whatever the protocol used) and US in patients with primary or recurrent hyperparathyroidism (all 23 studies considered in the review had included patients with primary hyperparathyroidism, and two (Amadou et al. [4] and Christakis et al. [31]) also included cases of recurrent hyperparathyroidism.

It is important to bear in mind that acquisition protocols for FCH PET/CT vary considerably. This can have an important impact on how images are interpreted and could bias their reported accuracy. FCH PET/CT was nonetheless superior to conventional scintigraphic or radiological approaches in most cases, regardless of the protocol used. In the majority of the studies considered, a single static acquisition was obtained at any time between 2 and 60 min after injecting the tracer. In 6/8 studies involving single static acquisitions, this interval ranged between 30 and 60 min. Full dynamic analysis could clarify the best timing of a static acquisition more precisely. This aspect does not seem to have been addressed in the literature to date and could be an interesting topic for future research. Only the paper by Prabhu et al. [15] reported the time active curves for parathyroid adenoma, thyroid gland and lymph node, demonstrating a higher uptake in parathyroid adenoma in the first 5 min after tracer injection. Michaud et al. [13] demonstrated that abnormal foci at a parathyroid gland were visible on early images, although the significant uptake in blood vessels imposed a more careful analysis of the cross-sectional images. Rep et al. [25] reported a slightly higher accuracy and sensitivity on scans obtained after 60 min than on those obtained after 5 min (94.1 vs. 96.5% and 90.5 vs. 93.6%, respectively). They consequently suggested that, for the preoperative localization of parathyroid gland, image acquisition was optimal 1 h after administering FCH.

Eight studies reported false positive results and 17 obtained false negative findings at 18F-Choline PET/CT (see Table 3), in accordance with the patient-based analysis. False-positive and false-negative results were frequently due to misinterpretation of thyroid anomalies, or due to a high uptake in normal or hyperplastic parathyroid glands, or in case of ectopic glands or adenomas with no specific characteristics or in case of very small adenomas with a fairly low number of oxyphilic cells.

As in the case of 99mTc-MIBI SPET/CT, some authors preferred to use a dual-phase FCH PET protocol (PET/CT or PET/MRI). It should be noted, however, that the kinetic characteristics of radiolabeled choline are very different from those of 99mTc-MIBI, and so the proper timing of the former cannot be deduced directly from the latter. 99mTc-MIBI accumulates more intensely in malignant cells because of their higher mitochondrial density and transmembrane electrical potential. Non-specific mechanisms lead to the uptake in nontumor cells with a greater metabolic activity or higher density of mitochondria—a situation encountered in atypical hyperplasia or particularly active tumor-like granulation. 99mTc-MIBI uptake in parathyroid foci was found to depend not on the cell type, but rather on either the size or the functional state of a lesion (Fukumoto et al. [35]). On the other hand, tumor cells with a high proliferation rate will have a high uptake of FCH to keep up with an increased demand for the synthesis of phospholipids (Vallabhajosula et al. [36]). A possible explanation for FCH uptake in benign parathyroid adenomas seems to be the increase in phospholipid-dependent choline kinase activity arising from PTH hypersecretion (Ishizuka et al. [37]).

A number of systematic reviews and meta-analyses on FCH PET/CT have been published in the last 2 years (Table 4) (Kim et al. [38]; Treglia et al. [39]; Boccalatte et al. [40]; Broos et al. [33]). In all cases, FCH PET/CT had an optimal performance in identifying benign parathyroid lesions. To the best of our knowledge, however, our systematic review is the first to include a large number of studies (*n* = 23), and to compare FCH PET/CT with conventional imaging (US and 99mTc-MIBI scanning), based on per-patient and per-lesion analyses. The present review also paid attention to the timing of image acquisition. In clinical practice, it seems that FCH PET/CT could be used for localizing and identifying benign parathyroid lesions, irrespective of the severity of PHPT (Beheshti et al. [29]), and particularly in patients with equivocal or negative conventional imaging.

**Table 4:** Summary of the published English systematic reviews about 18F-Choline PET/CT in hyperparathyroidism

Authors, ref	Year of pub	Meta-analysis	N of included studies	Comparison with other imaging	Outcome
Kim et al. [38]	2018	Yes	8	No	18F-Choline PET has a pooled sensitivity of 90% and a pooled specificity of 94% for the identification of HPT
Treglia et al. [39]	2019	Yes	18	No	Radiolabeled Choline PET has a pooled sensitivity of 95% and a pooled PPV of 91% for the identification of HPT
Boccalatte et al. [40]	2019	No	15	No	18F-Choline PET provides a high accuracy, sensitivity and specificity for the identification of HPT
Broos et al. [33]	2019	No	11	No	High detection rate of choline PET/CT in preoperative localization of hyperfunctioning parathyroid glands in patients with primary HPT

CT and MRI have always had a marginal role in parathyroid imaging, and been applied mainly when the results of US and 99mTc-MIBI are difficult to interpret, or when parathyroidectomy fails due to ectopic glands (Johnson et al. [41]). Recent technical advances enabling high-resolution MRI of the neck have increased the applicability of such techniques, however. Even small lesions can be reliably detected and characterized nowadays on conventional sequences or with methods like diffusion weighted imaging (DWI) (Yildiz et al. [42]). 4DCeCT combines standard multiplanar CT scanning (non-contrast, arterial and venous phases) with the fourth dimension of changes in contrast attenuation over time, providing both functional and anatomical information about the abnormal parathyroid gland. Some recent studies have reported the role of 4DCeCT in patients with persistent or recurrent parathyroid hyperfunction, showing a sensitivity until to 86% [43–46]. Piccardo et al. [21], showed that in 31 patients, the association of 18F-Choline PET with 4DCeCT could enhance the sensitivity to 100% in patients with persistent or recurrent hyperfunctioning parathyroid.

Argiro’ et al. [47], for instance, found MRI more sensitive (97.8%) than US or 99mTc-MIBI, either alone (89.1% and 83.6%, respectively) or combined (93.4%), for the pre-surgical detection of benign parathyroid lesions, as well as for the diagnosis of multiglandular disease and ectopic parathyroid adenomas.

Yildiz et al. [48] demonstrated that DWI enables solid parathyroid lesions to be distinguished from surrounding structures, and can also detect different types of lesions with peculiar MRI characteristics on T1w and T2w sequences. They found that parathyroid adenoma and hyperplasia usually appear as small lesions with well-defined margins and contrast enhancement, while parathyroid carcinomas are larger and less homogeneous.

Finally, in a recent study, Ozturk et al. [44] correctly localized 38 parathyroid lesions using 4D MRI, reporting a sensitivity of 90.5% and a PPV of 95%.

The advantages and disadvantages of all imaging techniques that can be used in the definition of parathyroid benign lesions are listed in Table 5.

**Table 5:** Pros and Cons of different imaging techniques used for parathyroid benign lesions

Imaging technique	Pros	Cons
US	No radiation exposure Widely available, cost effective Doppler can assist in distinguishing parathyroid lesions from other surrounding structures: identification of polar arteries of parathyroid glands vs. hilar blood supply of lymph nodes Concurrent assessment of the thyroid and possibility of performing percutaneous biopsies	Operator dependent Accuracy can be limited in patients with elevated body mass index Visualization of low inferior glands can be particularly difficult in patients unable to extend their neck Difficult detection of ectopic glands located in the mediastinum
MIBI SPECT	Widely available, cost effective Consolidated protocols described, including single-tracer double phase and dual-tracer single phase imaging Possibility of detecting ectopic lesions, particularly if in mediastinum	Thyroid nodules, thyroiditis and enlarged cervical lymph nodes may have delayed tracer washout and give the appearance of a thyroid adenoma Long acquisition time, compliance of patients is required Intermediate radiation dose (7–11 mSv)
4D-ceCT	Characterization of lesion enhancement could offer insights into the benign/malignant nature of the parathyroid lesion/s Excellent anatomic detail and possibility of detecting ectopic lesions	High radiation dose (10–27 mSv)
MRI	Characterization of lesion enhancement could offer insights into the benign/malignant nature of the parathyroid lesion/s Excellent anatomic detail and possibility of detecting ectopic lesions No radiation exposure	Long acquisition time, compliance of patients is required High costs and limited availability
18F-Choline PET/CT and PET/MR	Greater spatial resolution than MIBI SPECT and shorter image acquisition time	Lack of protocol standardization (optimal imaging timing, dynamic acquisition, administered activity etc.) Currently not clinically approved High costs and limited availability

In the light of the above, an approach combining FCH PET with MRI (using PET/MRI scanners or PET/CT with MRI) represents an optimal choice, improving on the accuracy of either method. Unfortunately, the paucity of data on PET/MRI in this field prevents us from drawing any further conclusions about the diagnostic potential of this technique.

## Conclusions

FCH PET is more accurate than conventional imaging modalities (US and 99mTc-MIBI SPET/CT, whatever the protocol used) in detecting benign parathyroid lesions. It has a potential role in both primary and recurrent hyperparathyroidism. Although it would be necessary to conduct a cost-effective analysis before adopting this imaging modality in clinical practice, it seems important to emphasize that, in selected cases, when the outcome of conventional US and scintigraphy is hard to interpret, FCH PET is an appropriate choice. PET/MRI is a very promising technique in this field, but further research is needed to fully assess its role.
